# Thromboembolic events in severe postpartum hemorrhage treated with recombinant activated factor VII: a systematic literature review and meta-analysis

**DOI:** 10.1016/j.rpth.2024.102533

**Published:** 2024-07-25

**Authors:** Johanna G. van der Bom, Frédéric J. Mercier, Damaris Bausch-Fluck, Mads Nordentoft, Morten Medici, Rezan Abdul-Kadir

**Affiliations:** 1 Department of Clinical Epidemiology, Leiden University Medical Center, Leiden, The Netherlands; 2 Department of Anaesthesia and Critical Care Medicine, A. Beclere Hospital - APHP, Paris-Saclay University, Clamart, France; 3 Novo Nordisk Health Care AG, Zurich, Switzerland; 4 Novo Nordisk A/S, Søborg, Denmark; 5 Department of Obstetrics and Gynaecology, The Royal Free National Health Service Foundation Hospital, London, United Kingdom; 6 Institute for Women's Health, University College London, London, United Kingdom

**Keywords:** drug-related side effects and adverse reactions, meta-analysis, postpartum hemorrhage, rFVIIa, safety, thromboembolism

## Abstract

Postpartum hemorrhage (PPH) is an obstetric complication with high associated morbidity. Recombinant activated factor VII (rFVIIa) is used to treat severe PPH when uterotonics fail to stop bleeding. However, data on the safety of rFVIIa treatment of severe PPH from adequately powered trials are lacking. We systematically reviewed published data on the incidence of thromboembolic events (TEs) in women with PPH treated or not treated with rFVIIa (PROSPERO CRD42022360736). Databases (Embase, MEDLINE, BIOSIS, Current Contents, and the Cochrane Library) were searched for peer-reviewed publications published between January 1996 and August 2022 and conference abstracts published between January 2017 and August 2022 using search terms related to thromboembolism or infarction and PPH. Data were extracted from all publications reporting on a general population of women with PPH with information on TEs. Descriptive summary statistics and the estimated proportion of TEs were analyzed using a generalized linear mixed model based on the binomial distribution. Quality assessments were based on the checklist by Downs and Black. From 1637 potentially eligible studies, 55 publications were included reporting on 611 women treated and 32,488 women not treated with rFVIIa. The global estimated proportion of TEs was 1.82% (prediction interval [PI], 0.30-10.23) and 0.72% (PI, 0.03-16.47) in women with severe PPH treated and those not treated with rFVIIa, respectively. The estimated proportions of TEs were similarly small, with wide and largely overlapping PIs. Additional well-designed trials are needed to improve understanding of TE incidence in PPH.

## Essentials

• Safety data on the use of recombinant activated factor (F)VII in postpartum hemorrhage from large trials are lacking.
• We systematically reviewed safety data on thromboembolic events in women with severe postpartum hemorrhage.
• The estimated proportion of thromboembolic events was 0.72% and 1.82% in women not treated and treated with recombinant activated FVII, respectively.
• Prediction intervals were wide and largely overlapping.

## 1 Background

Postpartum hemorrhage (PPH) is a potentially life-threatening complication that can occur during childbirth or shortly thereafter [1]. PPH can progress to severe PPH; however, the definition of severe PPH varies. Definitions include continuous blood loss of ≥1000 mL or ≥1500 mL within 24 hours, a drop in hemoglobin concentration of ≥4 g/dL, or the acute need for blood transfusions (≥4 units of red blood cells) [[1], [2], [3]]. PPH and severe PPH are estimated to occur in 6% to 11% and 1% to 3%, respectively, of all births globally [4]. The incidence and related maternal morbidity are higher in resource-poor countries compared with high-resource countries, although rates have recently been increasing in high-resource countries.

In 2022, recombinant activated factor VII (rFVIIa) was approved by the European Medicines Agency for the treatment of severe PPH when uterotonics fail to control bleeding [5]. Due to a previously observed 45% increase in the risk of arterial thromboembolic events (TEs) among patients without hemophilia treated off-label with rFVIIa, a discussion of related safety aspects is of interest, particularly the risk of developing a venous or arterial TE [6]. Venous TEs have been reported in patients with severe PPH who were treated with rFVIIa [7]. However, there is also an overall increased risk of thromboembolic complications during pregnancy and up to 6 weeks after delivery [5]. A higher TE risk is associated with high body mass index and multiple pregnancy. In women with PPH, the risk is further increased in combination with operative deliveries or when massive transfusions are required [[8], [9], [10]]. As such, a potentially causal relationship between rFVIIa administration and the occurrence of a TE cannot be easily determined [7].

Adequately powered trials that assess the safety of rFVIIa administration in women who experience severe PPH are lacking. Therefore, further studies are required to assess the potential contribution of rFVIIa to the thromboembolic risk associated with severe PPH. The systematic literature review presented here aims to summarize existing published data in order to estimate the incidence of arterial and venous TEs following PPH in women who were treated or not treated with rFVIIa. Hysterectomy rates among women treated or not treated with rFVIIa and hysterectomy rates before/after rFVIIa treatment among women exposed to rFVIIa were also evaluated.

## 2 Methods

### 2.1 Database search and screening strategy

This review was conducted in accordance with the principles outlined by Preferred Reporting Items for Systematic Reviews and Meta-Analysis (PRISMA) [11]. The protocol for this review has been published by PROSPERO, an international database of prospectively registered systematic reviews, and has been made available online (PROSPERO registration: CRD42022360736) [12]. Relevant databases (Embase, MEDLINE, BIOSIS, Current Contents, and the Cochrane Library) were searched in August 2022 using ProQuest. The search string used is outlined in Supplementary Table S1.

Peer-reviewed articles published between January 1996 and August 2022 and conference abstracts published between January 2017 and August 2022 were screened for eligibility using Covidence software (Veritas Health Innovation) [13]. The full details of the inclusion and exclusion criteria that were applied during the screening process are provided in Table 1. During this process, titles and abstracts were initially screened for inclusion, and in a second step, full texts of these publications were assessed for eligibility. Two reviewers independently performed the screening, and any disagreements were resolved by discussion or, if necessary, by a third reviewer.

**Table 1:** Inclusion and exclusion criteria.

Inclusion	Exclusion
Article in English	Only single patient in study (case report)
Article published between January 1996 and August 2022	Reports on cohorts selected based on safety outcome (TE or mortality)
Congress abstracts published between January 2017 and August 2022	Reports on women with acquired hemophilia
General population of women with PPH	Reports on abortions
RCT, consecutive cohort study (with ≥2 patients), retrospective/prospective single-center/multicenter study, or experimental trial	Review articles and guidelines
Reports with available data on TEs^a^	Reports on women with rare bleeding disorders
	Reports on nonclinical studies
	Reports on prevention of PPH
	Duplicate or overlapping reports (include latest report only)
	Reports with unclear or limited methodology or safety information
	Reports where safety information cannot be differentiated (ie, unclear if TEs are reported for patients exposed or not exposed to rFVIIa)

PPH, postpartum hemorrhage; RCT, randomized controlled trial; rFVIIa, recombinant activated factor VII; TE, thromboembolic event.

a Publications mentioning “no adverse events” were included if it was possible to deduce from the report that all thromboembolic events would have been included, if any.

### 2.2 Data extraction

One reviewer extracted the data and assessed the methodological quality of each publication according to a modified version of the checklist by Downs and Black [14]. The Downs and Black study quality assessment evaluates the methodology and reporting of both randomized and nonrandomized studies. Twenty-seven questions are grouped into sections on “reporting” (10 items), “external validity” (3 items), “internal validity—bias” (7 items), “internal validity—confounding” (6 items), and “power” (1 item) [14]. Studies were awarded 0 or 1 point for each question (2 points were possible for question 5). In question 27 of this checklist, 0 or 1 point was awarded depending on whether the study performed a power calculation, rather than rating the power of the study based on the sample size with 0–5 points. The highest possible score was 28. Studies that scored ≥15 points were classified as “higher quality” [15]. Extracted data and the methodological assessment were quality-checked by the second reviewer. Any unclarities were discussed with all authors to reach a consensus. Data extracted from each publication included publication information, study information, PPH definition and severity, intervention(s) described, total estimated blood loss, cause of PPH, mode of delivery, occurrence of TEs, and occurrence of hysterectomies. For publications that included women who were treated with rFVIIa, data on blood loss and hysterectomies before/after rFVIIa administration, bleeding response to rFVIIa treatment, and rFVIIa dosage were also extracted.

### 2.3 Statistical analysis

The primary analysis included descriptive summary statistics and an estimation of the proportion of all TEs, arterial TEs, and venous TEs in women with severe PPH (treated and not treated with rFVIIa). Women were grouped by severity of bleeding as classified in the original publication: severe, mixed (includes cases of both severe and less severe PPH), not classified (publication does not comment on severity). Descriptive summary statistics of the baseline characteristics are reported for included publications, tabulated by severity of PPH as defined in the original publication (severe, mixed, or not classified) and treatment group (treated or not treated with rFVIIa).

The analysis of the proportion of TEs in individual studies, grouped by geographical region, is presented in forest plots for women with severe PPH by treatment group (treated or not treated with rFVIIa). A meta-analysis of the TEs recorded across all publications was performed, and results are presented as raw and adjusted proportions together with the 95% prediction intervals (PIs), which were included due to the substantial heterogeneity of the data. The adjusted proportions of TEs, including 95% PI, were estimated using a generalized linear mixed model based on the binomial distribution with study as a random effect. All results were stratified by region: all regions, high-resource regions (Europe, North America, Australasia [Australia and New Zealand], and Japan), and resource-poor regions (South America, Africa, and Asia [excluding Japan]).

Robustness of the summary estimates was assessed using the heterogeneity measure *I*^*2*^, the trim-and-fill method, and the leave-one-out method. The statistical analyses were carried out with R using packages metafor and meta [[16], [17], [18]]. Additional information on the scripts used can be found in the Supplementary Methods. A sensitivity analysis considering study quality was performed. Studies were rated as of “higher quality” when scoring ≥15 points from a possible 28 points in total in the Downs and Black checklist, as has been reported previously [15]. This group of “higher-quality” studies was used for the sensitivity analyses.

## 3 Results

### 3.1 Screening results

**Figure 1:**
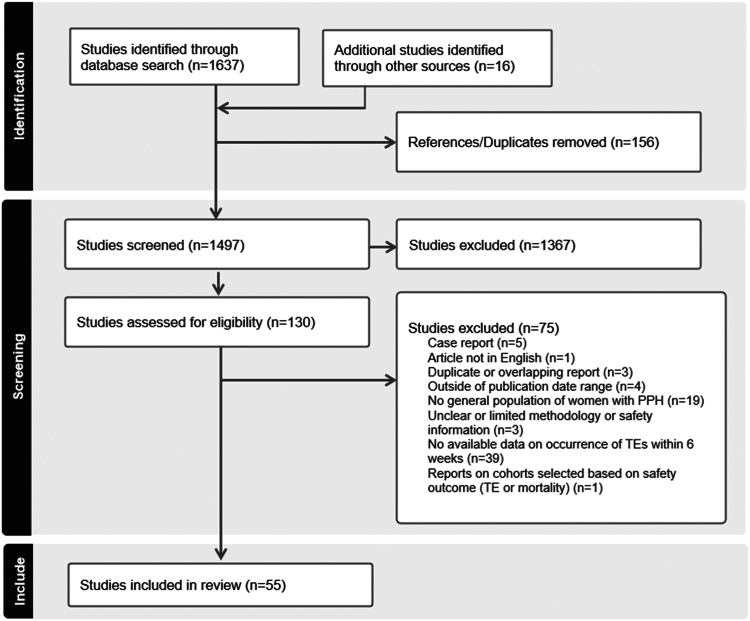
Preferred Reporting Items for Systematic Reviews and Meta-Analyses (PRISMA) flow diagram of study selection in the systematic literature review. PPH, postpartum hemorrhage; TE, thromboembolic event.

The search identified 1637 potentially relevant publications and the authors added another 16 references from other sources screened for inclusion. Any duplicates were removed, and all irrelevant references were excluded based on title and abstract according to the inclusion/exclusion criteria (Table 1). For this review, 130 full-text articles were assessed for eligibility as shown in the PRISMA flow diagram (Figure 1). Data were subsequently extracted from 55 studies [2,[19], [20], [21], [22], [23], [24], [25], [26], [27], [28], [29], [30], [31], [32], [33], [34], [35], [36], [37], [38], [39], [40], [41], [42], [43], [44], [45], [46], [47], [48], [49], [50], [51], [52], [53], [54], [55], [56], [57], [58], [59], [60], [61], [62], [63], [64], [65], [66], [67], [68], [69], [70], [71], [72]] (Supplementary Table S2). Of these, 15 publications reported data on women treated with rFVIIa (*n* = 611) and 44 publications reported data on women not treated with rFVIIa (*n* = 32,488). Data on both women treated and women not treated with rFVIIa were reported in 4 publications (*n* = 85 and *n* = 175, respectively). Fifty-one percent (28/55) of the included studies were rated as having “higher quality” based on the Downs and Black checklist.

### 3.2 Baseline characteristics

The mode of delivery was cesarean section in a mean of 56.8% (SD, 18.3%) of women with severe PPH treated with rFVIIa and 58.5% (SD, 21.8%) of women not treated with rFVIIa (Supplementary Table S3). Uterine atony was the most common cause of PPH (in women with severe PPH treated with rFVIIa, mean, 54.0%; SD, 20.2%; in women not treated with rFVIIa, mean, 57.6%; SD, 23.6%; Supplementary Table S3). Other frequent causes of PPH included abnormally invasive placenta and genital tract trauma (both in approximately one-fifth of women with severe PPH).

Hysterectomy was performed on average in 25.5% (SD, 20.3%) of women with severe PPH before the administration of rFVIIa and in 27.0% (SD, 17.2%) after treatment. The proportion of women treated with rFVIIa who underwent hysterectomy at any time, including publications in which the timing was not specified, was 50.5% (SD, 14.9%). Hysterectomy was reported in 24.1% (SD, 29.3%) of women who were not treated with rFVIIa. Information on blood loss and dosing of rFVIIa was reported inconsistently across the studies examined; however, typically, a single dose was administered (77.4% [SD, 22.1%] of all women with severe PPH when averaging proportion across publications). The hemostatic response was classified as adequate or better (including “reduced” or “stopped” bleeding) in 86.9% (SD, 8.2%) of women with severe PPH who received rFVIIa.

### 3.3 Meta-analysis results on TEs in severe PPH

**Figure 2:**
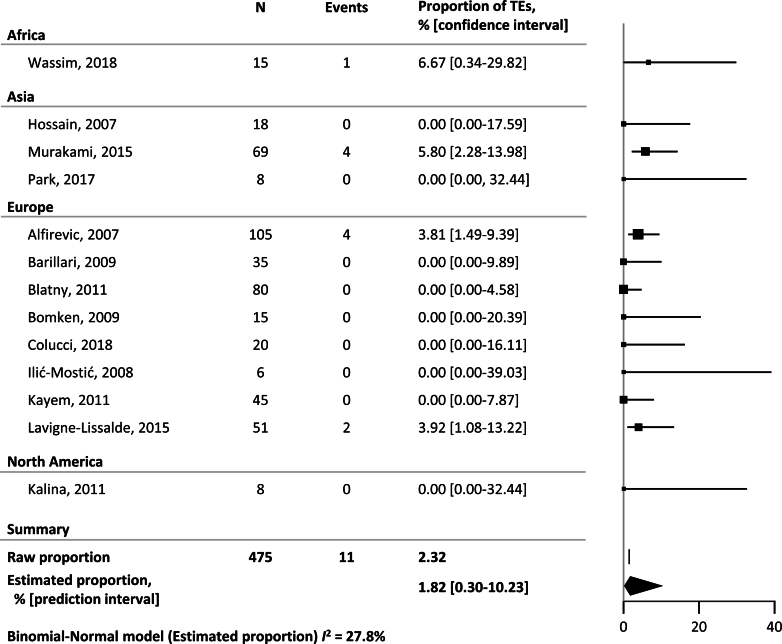
Meta-analysis of the proportion of thromboembolic events (TEs) in women with severe postpartum hemorrhage treated with recombinant activated factor VII. The size of the squares reflects the respective study size, while each line’s length denotes the CI. The width of the diamond reflects the prediction interval of the estimated proportion.

**Figure 3:**
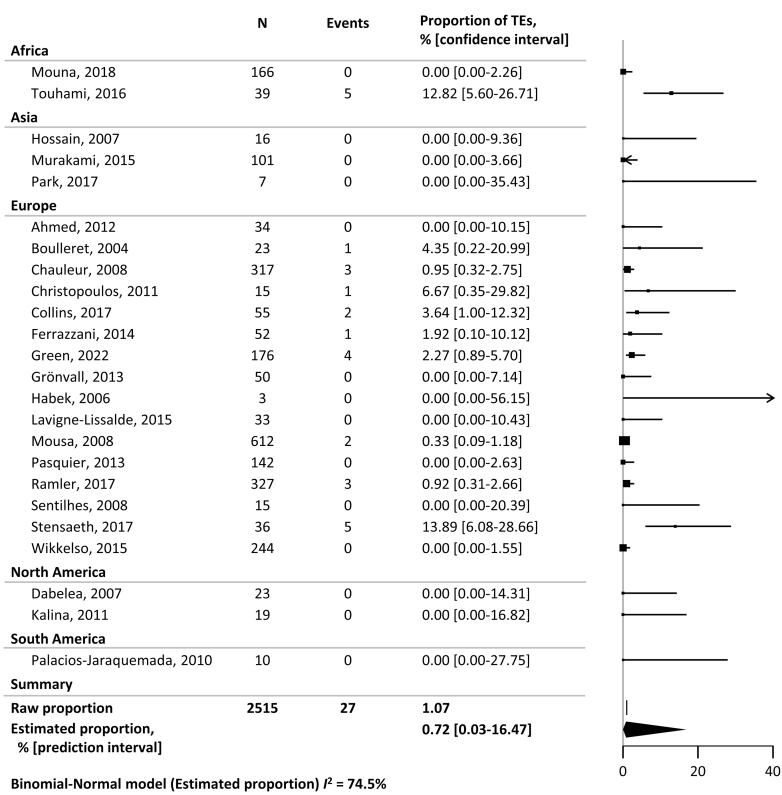
Meta-analysis of the proportion of thromboembolic events (TEs) in women with severe postpartum hemorrhage not treated with recombinant activated factor VII. The size of the squares reflects the respective study size, while each line’s length denotes the CI. Arrows indicate that the CI is wider than the x-axis. The width of the diamond reflects the prediction interval of the estimated proportion.

The estimated proportion of all TEs across all regions (high-resource and resource-poor) was 1.82% (95% PI, 0.30-10.23) in all women with severe PPH treated with rFVIIa and 0.72% (95% PI, 0.03-16.47) in women not treated with rFVIIa (Figures 2 and 3). An overview of the estimated proportions of TEs depending on bleeding severity is shown in Table 2. The estimated proportion of arterial TEs was 0.42% (95% PI, 0.09-1.94) in women with severe PPH treated with rFVIIa and 0.00% (95% PI, 0.00-70.41) in women not treated with rFVIIa. The estimated proportion of venous TEs was 1.45% (95% PI, 0.20-9.71) in women treated with rFVIIa and 0.51% (95% PI, 0.02-9.64) in women not treated with rFVIIa.

**Table 2:** Estimated proportion of thromboembolic events.

Severity of PPH	Not treated with rFVIIa			Treated with rFVIIa
	Severe	Mixed	Not classified	Severe
All TEs				
Included publications, *n* (%)	24 (100.0)	8 (80.0)	9 (90.0)	13 (100.0)
No. of women included	2515	20,761	2444	475
Estimated proportion (PI)	0.72 (0.03-16.47)	1.01 (0.08-11.20)	0.82 (0.49-1.37)	1.82 (0.30-10.23)
Arterial TEs				
Included publications, *n* (%)	22 (91.7)	6 (60.0)	5 (50.0)	13 (100.0)
No. of women included	2305	20,574	213	475
Estimated proportion (PI)	0.00 (0.00-70.41)	0.09 (0.05-0.17)	0	0.42 (0.09-1.94)
Venous TEs				
Included publications, *n* (%)	22 (91.7)	7 (70.0)	6 (60.0)	13 (100.0)
No. of women included	2305	26,574	263	475
Estimated proportion (PI)	0.51 (0.02-9.64)	0.53 (0.04-7.57)	0.29 (0.00-40.91)	1.45 (0.20-9.71)

Included publications report the proportion of publications included in the present analysis compared with the overall number of publications screened within each category.

PPH, postpartum hemorrhage; PI, prediction interval; rFVIIa, recombinant activated factor VII; TE, thromboembolic event.

Considering only publications from high-resource regions, the estimated proportion of all TEs was 1.66% (95% PI, 0.18-13.69) in those women treated with rFVIIa and 0.78% (95% PI, 0.05-11.56) in those not treated (Supplementary Table S4). Across all publications from resource-poor regions, the estimated proportion of all TEs was 2.44% (95% PI, 0.03-66.09) in women treated with rFVIIa and 0.18% (95% PI, 0.00-99.78) in women not treated with rFVIIa. The analysis was repeated with only “higher-quality” publications (scoring 15 points or more using the Downs and Black study quality assessment; Supplementary Table S4). Within this subanalysis, the estimated proportion of all TEs was 0.25% (95% PI, 0.00-49.39) for women treated with rFVIIa (*n* = 286) and 0.80% (95% PI, 0.03-18.98) for women who were not treated with rFVIIa (*n* = 1555).

## 4 Discussion

### 4.1 Main findings and interpretation

This systematic literature review included a meta-analysis of currently available safety data regarding TEs gathered from published studies on the use of rFVIIa in severe PPH. Data were extracted in a systematic manner from publications on women with PPH that met the inclusion and exclusion criteria.

The main objective of our analysis was to summarize the available published data regarding TEs. In women with severe PPH, the estimated proportion of all TEs among women treated with rFVIIa was 1.82% and that among women not treated with rFVIIa was 0.72%. When the analysis only included “higher-quality” publications, the estimated proportion of all TEs was 0.25% and 0.80% in women treated and women not treated with rFVIIa, respectively. The response to treatment with rFVIIa was rated as adequate or better on average in 4 out of 5 women with severe PPH. This is broadly consistent with previous publications summarizing the use of rFVIIa in PPH [73]. From a clinical perspective, the estimated proportions of TEs from the present study are relatively small. However, there were wide and largely overlapping PIs for all analyses, limiting the ability to make interpretations. As with any intervention, the treatments available for severe PPH (transfusion, pharmacological, mechanical, and surgical options) are known to have associated risks for adverse events. In addition to procedure-related risks, the TE risk can be increased when invasive interventions are performed. Therefore, the risk of TEs with rFVIIa treatment should be considered within this context and its use should also be assessed on a case-by-case basis.

Other studies have reviewed the incidence of TEs in people treated with rFVIIa for off-label indications. In a broad population treated with rFVIIa for various off-label indications in double-blind, placebo-controlled randomized controlled trials, the TE rate was 10.2% among rFVIIa-treated patients versus 8.7% among placebo-treated patients [74]. Venous TEs were similar between patients receiving rFVIIa (5.3%) versus placebo (5.7%), but arterial TEs were higher in patients who received rFVIIa (5.5%) than in those who received placebo (3.2%), especially in those over 65 years of age [74]. In contrast, results from our study showed an estimated proportion of TEs under 3% for all analyses and did not show an increase in arterial TEs in patients receiving rFVIIa compared with those not treated with rFVIIa, although with large PIs. The population of our study was more homogeneous, it included young women giving birth who experienced PPH. Due to the emergency and life-threatening situation of severe PPH, the use of placebo-controlled and double-blinded studies is generally unrealistic.

The results from the present study do not suggest a clinically relevant increased incidence of TEs in patients with severe PPH treated with rFVIIa. However, it must be noted that the wide and overlapping PIs preclude definitive conclusions. Further understanding of the incidence of TEs within the population of women with PPH, both treated and untreated with rFVIIa, will be aided by additional data collection and future analyses.

### 4.2 Limitations

There are a number of limitations that need to be considered for this study. First, patients selected for treatment with rFVIIa differ from those not treated with rFVIIa. Women who were treated with rFVIIa might have had a more severe clinical condition than women who were not treated with rFVIIa and may therefore have been at higher risk of developing a TE. Indeed, current treatment guidelines generally recommend the use of rFVIIa as a last resort, before or after hysterectomy. In the present analysis, the mean hysterectomy rate at any time during treatment of PPH in women not treated with rFVIIa was 24.1%. In contrast, 50.5% of women treated with rFVIIa had a hysterectomy, more than half of whom had the hysterectomy prior to rFVIIa administration, a last-resort procedure that has been reported to be associated with a higher risk for venous TEs [75]. In addition, there is no common standard in the definition of PPH severity. Consequently, individual publications may have used different bleeding thresholds for defining PPH severity, leading to heterogeneity between the study populations included in our review.

Furthermore, the number of TEs identified and reported depends on the methods applied to each publication, introducing potential measurement bias to the current analysis. There were also large variations in the interventions used to control PPH and the comprehensiveness of follow-up. Follow-up times to record the occurrence of any TEs varied from a few days (usually until discharge) to up to 6 weeks or more. It is conceivable that studies reporting off-label use of rFVIIa may have performed longer or more thorough monitoring of adverse TEs due to its procoagulant mechanism of action, introducing potential reporting bias.

Many factors contribute to the risk of developing a TE. These include massive transfusion; cesarean section; hemostatic surgery, notably hysterectomy; obesity; and bacterial infection. Conversely, thromboprophylaxis may substantially reduce the risk of a TE, and it can be assumed that this measure is not provided uniformly across all geographic regions despite guidance in current clinical practice recommendations. The factors listed above were not always described in sufficient detail in all publications included here. As a consequence, the (in)comparability between groups or publications cannot be assessed comprehensively.

### 4.3 Future recommendations

Large, well-designed studies on the incidence of TEs in PPH are urgently needed. In order to improve the comparability of data generated by future studies, the baseline and time-dependent clinical characteristics and outcomes of study populations need to be systematically assessed. Establishing a widely accepted definition of PPH and severe PPH would allow better classification of patient cohorts. The follow-up times for the recording of adverse events and the assessment method used should be clearly defined and follow-up time, where possible, should be extended to 6 weeks.

Future meta-analyses may be able to draw on a greater number of high-quality studies documenting the incidence of TEs in severe PPH with or without treatment with rFVIIa. The present study can serve as a template for such analyses. It has been conducted according to PRISMA principles and the full protocol is available at PROSPERO [11,12].

### 5 Conclusion

Taken together, the estimated proportions of all TEs for women treated and women not treated with rFVIIa were similarly small with wide, largely overlapping PIs. Future analyses based on large, well-designed clinical studies are required to reveal more accurate incidences of TEs in specified subgroups of severe PPH.
