# Supplementary material for: Thromboembolic events in severe postpartum hemorrhage treated with recombinant activated factor VII: a systematic literature review and meta-analysis
Source: Res Pract Thromb Haemost. 2024 Jul 25;8(5):102533. doi: 10.1016/j.rpth.2024.102533 (PMC11387238; doi:10.1016/j.rpth.2024.102533)
Supplement: Supplementary material [file mmc1.docx]

Thromboembolic events in severe postpartum haemorrhage treated with recombinant activated factor VII: a systematic literature review and meta-analysis

Johanna van der Bom^1^*, Frédéric J. Mercier^2^, Damaris Bausch-Fluck^3^, Mads Nordentoft^4^, Morten Medici^4^, Rezan Abdul-Kadir^5,6^

*^1^Department of Clinical Epidemiology, Leiden University Medical Center, Leiden, The Netherlands; ^2^Department of Anaesthesia and Critical Care Medicine, A. Beclere hospital - APHP, Paris-Saclay University, Clamart, France; ^3^Novo Nordisk Health Care AG, Zurich, Switzerland; ^4^Novo Nordisk A/S, Søborg, Denmark; ^5^Department of Obstetrics and Gynaecology, The Royal Free National Health Service Foundation Hospital, London, United Kingdom; ^6^Institute for Women's Health, University College London, London, United Kingdom.*

*Corresponding author

**Supplementary Methods**

**Script for estimating proportions**

# This script demonstrates how the proportions were estimated

# Used with R version 4.2.2 and metafor version 3.8.1

# Script by Morten Medici (2023)

# The example data below corresponds to the data presented in the paper for the following group:

# All regions, severe PPH, counting all thrombotic events, for women not treated with rFVIIa, any paper quality

pop <- c(142, 317, 15, 10, 166, 176, 39, 36,

23, 327, 23, 19, 101, 15, 244, 7,

33, 612, 3, 34, 50, 52, 16, 55)

n_events <- c(0, 3, 0, 0, 0, 4, 5, 5,

1, 3, 0, 0, 0, 1, 0, 0,

0, 2, 0, 0, 0, 1, 0, 2)

id <- seq(length(pop))

dat <- data.frame(id = id,

pop = pop,

n_events = n_events)

# Fit random effects binomial-normal model using package metafor

res_glmm <- metafor::rma.glmm(

xi=n_events, ni=pop, data=dat,

measure="PLO", # Logit transformed proportion

method="ML", # Fit a random effects model

nAGQ=10, # Notice this is higher than the default

test="t", # Using t-test as recommended by Riley, Higgins, Deeks (2011), doi:10.1136/bmj.d549

# Implemented in metafor using df = N_observations - 1

# as discussed in Stijnen, Hamza, Özdemir (2010), doi: 10.1002/sim.4040

level=95 # Confidence interval level set to 95%

)

# Get predictions intervals on the correct scale (absolute proportions)

pred.glmm <- predict(res_glmm, transf=metafor::transf.ilogit, digits=4)

# Calculate raw proportions as a crude average

pred.raw <- sum(dat$n_events)/sum(dat$pop)

**Supplementary Table S1.** Search strategy.

| Set# | Search for: |
| --- | --- |
| S1 | **MESH**.EXACT.EXPLODE("Embolism and Thrombosis") OR **MESH**.EXACT.EXPLODE("Myocardial Infarction") OR **EMB**.EXACT.EXPLODE("thromboembolism") OR **EMB**.EXACT.EXPLODE("cerebrovascular accident") OR **EMB**.EXACT.EXPLODE("heart infarction") OR **EMB**.EXACT.EXPLODE("brain infarction") |
| S2 | Ti,ab(embolic or emboli or embolism or thrombosis or thromboemboli* or thrombi or thrombus  or thrombotic or stroke or infarction or ischemi* or “cerebrovascular accident” or thrombophlebitis) |
| S3 | S2 OR S1 |
| S4 | MJEMB.EXACT("postpartum hemorrhage") OR MJMESH.EXACT("Postpartum Hemorrhage") or ti,ab((Postpartum or post-partum or partum or obstetric* or *birth or caesarean or caesarean) near/3 ("excessive bleeding" or "massive bleeding" or "severe bleeding“ or h*emorrhag*)) |
| S5 | S4 AND S3 |
| S6 | S5 AND (la.exact("ENG")) |

**Supplementary Table S2**. Included studies.

| **Study** | **QA score** | **N**  **Treated/not treated with rFVIIa** | **Novo Nordisk**  **sponsorship** | **Geographical region** |
| --- | --- | --- | --- | --- |
| Ahmed, 2012 [1] | 19 | 0/34 | - | Europe |
| Alfirevic, 2007 [2] | 14 | 108/0 | - | Europe |
| Barillari, 2009 [3] | 15 | 35/0 | Publication support | Europe |
| Blatny, 2011 [4] | 9 | 80/0 | Registry support | Europe |
| Bomken, 2009 [5] | 15 | 15/0 | - | Europe |
| Bouet, 2016 [6] | 21 | 0/289 | - | Europe |
| Boulleret, 2004 [7] | 11 | 0/36 | - | Europe |
| Chauleur, 2008 [8] | 16 | 0/317 | - | Europe |
| Christopoulos, 2011 [9] | 11 | 0/15 | - | Europe |
| Collins, 2017 [10] | 26 | 0/55 | - | Europe |
| Colucci, 2018 [11] | 18 | 20/0 | - | Europe |
| Dabelea, 2007 [12] | 10 | 0/23 | - | North America |
| Ducloy-Bouthors, 2011 [13] | 26 | 0/144 | - | Europe |
| Ducloy-Bouthors, 2021 [14] | 25 | 0/437 | - | Europe |
| Escobar, 2019 [15] | 16 | 0/142 | - | South America |
| Fatima, 2018 [16] | 10 | 0/50 | - | Asia |
| Ferrazzani, 2014 [17] | 11 | 0/52 | - | Europe |
| Green, 2022 [18] | 21 | 0/180 | - | Europe |
| Grönvall, 2013 [19] | 12 | 0/50 | - | Europe |
| Habek, 2006 [20] | 8 | 0/3 | - | Europe |
| Hossain, 2007 [21] | 18 | 18/16 | - | Asia |
| Ilić-Mostić, 2008 [22] | 9 | 6/0 | - | Europe |
| James, 2009 [23] | 10 | 0/108 | - | North America |
| Kalina, 2011 [24] | 15 | 8/19 | - | North America |
| Kallianidis, 2021 [25] | 10 | 0/1302 | - | Europe |
| Kamijo, 2022 [26] | 12 | 0/143 | - | Asia |
| Kavak, 2014 [27] | 11 | 0/7 | - | Europe |
| Kayem, 2011 [28] | 16 | 48/0 | - | Europe |
| Kwong, 2020 [29] | 18 | 0/43 | - | Asia |
| Lavigne-Lissalde, 2015 [30] | 19 | 51/33 | - | Europe |
| Lim, 2018 [31] | 18 | 0/28 | - | North America |
| Makino, 2015 [32] | 11 | 0/101 | - | Asia |
| Mouna, 2018 [33] | 13 | 0/166 | - | Africa |
| Mousa, 2008 [34] | 18 | 0/612 | - | Europe |
| Murakami, 2015 [35] | 12 | 69/0 | - | Asia |
| Obata, 2017 [36] | 17 | 0/28 | - | Asia |
| Palacios-Jaraquemada, 2010 [37] | 13 | 0/10 | - | South America |
| Park, 2017 [38] | 18 | 8/7 | - | Asia |
| Pasquier, 2013 [39] | 17 | 0/142 | - | Europe |
| Pellerin, 2013 [40] | 16 | 0/44 | - | Europe |
| Phillips, 2009 [41] | 15 | 105/0 | Registry support | Australasia |
| Phillips, 2020 [42] | 10 | 0/45 | - | North America |
| Ramler, 2017 [43] | 14 | 0/327 | - | Europe |
| Ramler, 2019 [44] | 20 | 0/455 | - | Europe |
| Sentilhes, 2008 [45] | 14 | 0/15 | - | Europe |
| Sharma, 2021 [46] | 11 | 0/53 | - | Asia |
| Sobieszczyk, 2006 [47] | 13 | 25/0 | Registry support | Europe |
| Stein, 2019 [48] | 12 | 0/26 | - | Europe |
| Stensaeth, 2017 [49] | 14 | 0/36 | - | Europe |
| Thurn, 2018 [50] | 19 | 0/6500 | - | Europe |
| Touhami, 2016 [51] | 20 | 0/39 | - | Africa |
| Wassim, 2018 [52] | 11 | 15/0 | - | Africa |
| Wikkelso, 2015[53] | 26 | 0/244 | - | Europe |
| WOMAN trial collaborators, 2017 [54] | 26 | 0/20,060 | - | Global |
| Zainab, 2015 [55] | 9 | 0/52 | - | Asia |

QA was performed according to the modified Downs and Black checklist [56].

N, number; PPH, postpartum haemorrhage; QA, quality assessment.

**Supplementary Table S3**. Baseline information of included women.

|  | **Not treated with rFVIIa** | | | **Treated with rFVIIa** | | |
| --- | --- | --- | --- | --- | --- | --- |
| **Severity of PPH** | **Severe** | **Mixed** | **Not classified** | **Severe** | **Mixed** | **Not classified** |
| **Included publications, N (%)** | 24 (100.0) | 10 (100.0) | 10 (100.0) | 13 (100.0) | 1 (100.0) | 1 (100.0) |
| **Number of women included** | 2,532 | 27,356 | 2,600 | 481 | 25 | 105 |
| Mean (SD) | 105.5 (144.6) | 2,735.6 (6,411.4) | 260 (402.1) | 37 (32.3) | 25 (-) | 105 (-) |
| Median (IQR) | 37.5 (18.2–148) | 97.5 (46.8–252.8) | 79 (32.0–363.5) | 20 (15–51) | 25 (25–25) | 105 (105–105) |
| **Mode of delivery** | | | | | | |
| **Proportion of women, CS (%)** | | | | | | |
| Included publications, N (%) | 16 (66.7) | 8 (80.0) | 9 (90.0) | 9 (69.2) | 1 (100.0) | - |
| Mean (SD) | 58.5 (21.8) | 25.8 (23.9) | 49 (39.5) | 56.8 (18.3) | 58.3 (-) | - |
| Median (IQR) | 62.4 (46.1–73.1) | 28.8 (0–40.9) | 54.6 (20.5–71.4) | 54.3 (50–55.6) | 58.3 (58.3–58.3) | - |
| **Proportion of women, VD (%)** | | | | | | |
| Included publications, N (%) | 16 (66.7) | 7 (70.0) | 8 (80.0) | 9 (69.2) | 1 (100.0) | - |
| Mean (SD) | 41.5 (21.8) | 75.7 (25.3) | 53.8 (41.3) | 48.8 (8.5) | 41.7 (-) | - |
| Median (IQR) | 37.6 (26.9–53.9) | 71.4 (58.5–100) | 61 (21.4–84.7) | 48.4 (45.2–50) | 41.7 (41.7–41.7) | - |
| **Cause of PPH** | | | | | | |
| **Proportion of women, trauma (%)** | | | | | | |
| Included publications, N (%) | 16 (66.7) | 5 (50.0) | 7 (70.0) | 8 (61.5) | - | 1 (100.0) |
| Mean (SD) | 18 (16.6) | 16.8 (22.2) | 15.7 (17.7) | 20.9 (16.8) | - | 6.7 (-) |
| Median (IQR) | 13.7 (5.8–30.6) | 6.7 (4.2­–18.6) | 10.5 (6.3–16.9) | 19.2 (7–35) | - | 6.7 (6.7–6.7) |
| **Proportion of women, uterine atony (%)** | | | | | | |
| Included publications, N (%) | 18 (75.0) | 6 (60.0) | 6 (60.0) | 8 (61.5) | - | 1 (100.0) |
| Mean (SD) | 57.6 (23.6) | 59.7 (27.7) | 71.7 (22) | 54 (20.2) | - | 18.1 (-) |
| Median (IQR) | 58 (43–76.3) | 57.7 (46.8–83.2) | 77.8 (58.3–81.8) | 46.7 (41.5–60.4) | - | 18.1 (18.1–18.1) |
| **Proportion of women, AIP (%)** | | | | | | |
| Included publications, N (%) | 16 (66.7) | 5 (50.0) | 8 (80.0) | 8 (61.5) | - | 1 (100.0) |
| Mean (SD) | 19.5 (19) | 17.2 (8.3) | 28.9 (44.6) | 16.3 (7.1) | - | 28.6 (-) |
| Median (IQR) | 15.8 (10.2–20.4) | 18.2 (9.5–21.1) | 4 (0–42.6) | 18.3 (16.1–20.1) | - | 28.6 (28.6–28.6) |
| **Proportion of women, placental abruption (%)** | | | | | | |
| Included publications, N (%) | 14 (58.3) | 3 (30.0) | 7 (70.0) | 8 (61.5) | - | 1 (100.0) |
| Mean (SD) | 5.7 (8.8) | 2.9 (4.9) | 4.6 (6.3) | 4.2 (6.3) | - | 8.6 (-) |
| Median (IQR) | 3.8 (0–7.6) | 0 (0–4.3) | 0.7 (0–8.5) | 0 (0–7.5) | - | 8.6 (8.6–8.6) |
| **Proportion of women, placental retention (%)** | | | | | | |
| Included publications, N (%) | 15 (62.5) | 5 (50.0) | 6 (60.0) | 8 (61.5) | - | 1 (100.0) |
| Mean (SD) | 13.7 (17.9) | 3 (3.1) | 12.1 (9.1) | 6.6 (10) | - | 3.8 (-) |
| Median (IQR) | 5.9 (0–24.9) | 3.5 (0–3.8) | 11 (8–14.8) | 0 (0–11.8) | - | 3.8 (3.8–3.8) |
| **Proportion of women, other causes (%)** | | | | | | |
| Included publications, N (%) | 17 (70.8) | 6 (60.0) | 5 (50.0) | 8 (61.5) | - | 1 (100.0) |
| Mean (SD) | 11.1 (12.2) | 14.4 (18.5) | 14.4 (18.6) | 15.4 (10.1) | - | 34.3 (-) |
| Median (IQR) | 8.2 (0–20) | 34.3 (7.4–41.5) | 4.5 (2.1–21.4) | 15.3 (8.8–21.5) | - | 34.3 (34.3–34.3) |
| **Clinical outcome** | | | | | | |
| **Proportion of women, hysterectomy anytime (%)** | | | | | | |
| Included publications, N (%) | 22 (91.7) | 5 (50) | 7 (70.0) | 11 (84.6) | 1 (100.0) | 1 (100.0) |
| Mean (SD) | 24.1 (29.3) | 22.8 (43.4) | 20.6 (35.5) | 50.5 (14.9) | 48 (-) | 53.3 (-) |
| Median (IQR) | 14.9 (2.8–32.7) | 1.9 (1.4–10.7) | 7.7 (3.3–15) | 52.1 (45.1–58.1) | 48 (48–48) | 53.3 (53.3–53.3) |

Included publications (%) report the proportion of publications included in the present analysis compared with the overall number of publications screened within each category.

AIP, abnormally invasive placenta; CS, caesarean section; IQR, interquartile range; N, number; PPH, postpartum haemorrhage; rFVIIa, recombinant activated factor VII; SD, standard deviation; VD, vaginal delivery

**Supplementary Table S4.** Estimated proportion of thromboembolic events in sensitivity analyses.

|  | **Not treated with rFVIIa** | | | **Treated with rFVIIa** |
| --- | --- | --- | --- | --- |
| **Severity of PPH** | **Severe** | **Mixed** | **Not classified** | **Severe** |
| **“Higher-quality” studies** | | | | |
| **All TEs** | | | | |
| Included publications, N (%) | 12 (50.0) | 7 (70.0) | 3 (30.0) | 10 (76.9) |
| Number of women included | 1,555 | 20,716 | 920 | 286 |
| Estimated proportion, (PI) | 0.80 (0.03-18.98) | 1.11 (0.08-13.48) | 0.54 (0.08-3.63) | 0.25 (0.00-49.39) |
| **Arterial TEs** | | | | |
| Included publications, N (%) | 10 (41.7) | 6 (60.0) | - | 10 (76.9) |
| Number of women included | 1,345 | 20,574 | - | 286 |
| Estimated proportion, (PI) | 0.028 (0-28.41) | 0.09 (0.05-0.17) | - | -* |
| **Venous TEs** | | | | |
| Included publications, N (%) | 10 (41.7) | 7 (70.0) | - | 10 (76.9) |
| Number of women included | 1,345 | 26,574 | - | 286 |
| Estimated proportion, (PI) | 0.58 (0.01-23.58) | 0.53 (0.04-7.57) | - | 0.25 (0.00-49.39) |
| **High-resource regions** | | | | |
| **All TEs** | | | | |
| Included publications, N (%) | 19 (79.2) | 5 (50.0) | 9 (90.0) | 10 (76.9) |
| Number of women included | 2,277 | 506 | 2,444 | 434 |
| Estimated proportion, (PI) | 0.78 (0.05-11.56) | 0.73 (0.05-9.27) | 0.82 (0.49-1.37) | 1.66 (0.18-13.69) |
| **Arterial TEs** | | | | |
| Included publications, N (%) | 17 (70.8) | 3 (30.0) | 5 (50.0) | 10 (76.9) |
| Number of women included | 2,067 | 461 | 213 | 434 |
| Estimated proportion, (PI) | 0.01 (0.00-75.08) | -* | -* | 0.23 (0.02-2.18) |
| **Venous TEs** | | | | |
| Included publications, N (%) | 17 (70.8) | 4 (40.0) | 5 (50.0) | 10 (76.9) |
| Number of women included | 2,067 | 6,461 | 213 | 434 |
| Estimated proportion, (PI) | 0.58 (0.32-1.07) | 0.39 (0.01-10.25) | 0.37 (0.00-42.32) | 1.76 (0.32-9.14) |
| **Resource-poor regions** | | | | |
| **All TEs** | | | | |
| Included publications, N (%) | 5 (20.8) | 4 (40) | - | 3 (23.1) |
| Number of women included | 238 | 20,255 | - | 41 |
| Estimated proportion, (PI) | 0.18 (0.00-99.78) | 1.45 (0.03-43.71) | - | 2.44 (0.03-66.09) |
| **Arterial TEs** | | | | |
| Included publications, N (%) | 5 (20.8) | 3 (30.0) | - | 3 (23.1) |
| Number of women included | 238 | 20,113 | - | 41 |
| Estimated proportion, (PI) | - | 0.09 (0.04-0.25) | - | 2.44 (0.03-66.09) |
| **Venous TEs** | | | | |
| Included publications, N (%) | 5 (20.8) | 3 (30.0) | - | 3 (23.1) |
| Number of women included | 238 | 20,113 | - | 41 |
| Estimated proportion, (PI) | 0.18 (0.00-99.78) | 0.82 (0.00-78.29) | - | -* |

Included publications (%) report the proportion of publications in each category included in the present analysis compared with the overall number of publications screened within each category.

*No events were recorded; therefore no linear mixed model fit was conducted.

N, number; PPH, postpartum haemorrhage; PI, prediction interval; rFVIIa, recombinant activated factor VII; TE, thromboembolic event

**References**

[1] Ahmed S, Harrity C, Johnson S, Varadkar S, McMorrow S, Fanning R, et al. The efficacy of fibrinogen concentrate compared with cryoprecipitate in major obstetric haemorrhage--an observational study. Transfus Med. 2012;22:344-9. <https://doi.org/10.1111/j.1365-3148.2012.01178.x>

[2] Alfirevic Z, Elbourne D, Pavord S, Bolte A, Van Geijn H, Mercier F, et al. Use of recombinant activated factor VII in primary postpartum hemorrhage: the Northern European registry 2000-2004. Obstet Gynecol. 2007;110:1270-8. <https://doi.org/10.1097/01.Aog.0000288515.48066.99>

[3] Barillari G, Frigo MG, Casarotto M, Farnia A, Massè B, Wetzl R, et al. Use of recombinant activated factor VII in severe post-partum haemorrhage: data from the Italian Registry: a multicentric observational retrospective study. Thromb Res. 2009;124:e41-7. <https://doi.org/10.1016/j.thromres.2009.08.018>

[4] Blatny J, Seidlova D, Penka M, Ovesna P, Brabec P, Sevcik P, et al. Severe postpartum haemorrhage treated with recombinant activated factor VII in 80 Czech patients: analysis of the UniSeven registry. Int J Obstet Anesth. 2011;20:367-8. <https://doi.org/10.1016/j.ijoa.2011.07.008>

[5] Bomken C, Mathai S, Biss T, Loughney A, Hanley J. Recombinant activated factor VII (rFVIIa) in the management of major obstetric haemorrhage: a case series and a proposed guideline for use. Obstet Gynecol Int. 2009;2009:364843. <https://doi.org/10.1155/2009/364843>

[6] Bouet PE, Ruiz V, Legendre G, Gillard P, Descamps P, Sentilhes L. Policy of high-dose tranexamic acid for treating postpartum hemorrhage after vaginal delivery. J Matern Fetal Neonatal Med. 2016;29:1617-22. <https://doi.org/10.3109/14767058.2015.1056731>

[7] Boulleret C, Chahid T, Gallot D, Mofid R, Tran Hai D, Ravel A, et al. Hypogastric arterial selective and superselective embolization for severe postpartum hemorrhage: a retrospective review of 36 cases. Cardiovasc Intervent Radiol. 2004;27:344-8. <https://doi.org/10.1007/s00270-003-2698-6>

[8] Chauleur C, Cochery-Nouvellon E, Mercier E, Aya G, Marès P, Mismetti P, et al. Analysis of the venous thromboembolic risk associated with severe postpartum haemorrhage in the NOHA First cohort. Thromb Haemost. 2008;100:773-9. <https://doi.org/10.1160/TH08-06-0376>

[9] Christopoulos P, Hassiakos D, Tsitoura A, Panoulis K, Papadias K, Vitoratos N. Obstetric hysterectomy: a review of cases over 16 years. J Obstet Gynaecol. 2011;31:139-41. <https://doi.org/10.3109/01443615.2010.536858>

[10] Collins PW, Cannings-John R, Bruynseels D, Mallaiah S, Dick J, Elton C, et al. Viscoelastometric-guided early fibrinogen concentrate replacement during postpartum haemorrhage: OBS2, a double-blind randomized controlled trial. Br J Anaesth. 2017;119:411-21. <https://doi.org/10.1093/bja/aex181>

[11] Colucci G, Helsing K, Biasiutti FD, Raio L, Schmid P, Tsakiris DA, et al. Standardized management protocol in severe postpartum hemorrhage: a single-center study. Clin Appl Thromb Hemost. 2018;24:884-93. <https://doi.org/10.1177/1076029618758956>

[12] Dabelea V, Schultze PM, McDuffie RS, Jr. Intrauterine balloon tamponade in the management of postpartum hemorrhage. Am J Perinatol. 2007;24:359-64. <https://doi.org/10.1055/s-2007-984402>

[13] Ducloy-Bouthors AS, Jude B, Duhamel A, Broisin F, Huissoud C, Keita-Meyer H, et al. High-dose tranexamic acid reduces blood loss in postpartum haemorrhage. Crit Care. 2011;15:R117. <https://doi.org/10.1186/cc10143>

[14] Ducloy-Bouthors AS, Mercier FJ, Grouin JM, Bayoumeu F, Corouge J, Le Gouez A, et al. Early and systematic administration of fibrinogen concentrate in postpartum haemorrhage following vaginal delivery: the FIDEL randomised controlled trial. BJOG. 2021;128:1814-23. <https://doi.org/10.1111/1471-0528.16699>

[15] Escobar MF, Suso JP, Hincapié MA, Echavarría MP, Fernández P, Carvajal J. Experience of combined use of a Bakri uterine balloon and a non-pneumatic anti-shock garment in a university hospital in Colombia. Int J Gynaecol Obstet. 2019;146:244-9. <https://doi.org/10.1002/ijgo.12872>

[16] Fatima T, Kousar S, Mohsin B, Tabassum Z. Effectiveness of single compression suture in management of atonic uterus during C-section. PJMHS. 2018;12:681-3.

[17] Ferrazzani S, Iadarola R, Perrelli A, Botta A, Moresi S, Salvi S, et al. Use of an intrauterine inflated catheter balloon in massive post-partum hemorrhage: a series of 52 cases. J Obstet Gynaecol Res. 2014;40:1603-10. <https://doi.org/10.1111/jog.12404>

[18] Green L, Daru J, Gonzalez Carreras FJ, Lanz D, Pardo MC, Pérez T, et al. Early cryoprecipitate transfusion versus standard care in severe postpartum haemorrhage: a pilot cluster-randomised trial. Anaesthesia. 2022;77:175-84. <https://doi.org/10.1111/anae.15595>

[19] Grönvall M, Tikkanen M, Tallberg E, Paavonen J, Stefanovic V. Use of Bakri balloon tamponade in the treatment of postpartum hemorrhage: a series of 50 cases from a tertiary teaching hospital. Acta Obstet Gynecol Scand. 2013;92:433-8. <https://doi.org/10.1111/j.1600-0412.2012.01531.x>

[20] Habek D, Kulas T, Bobić-Vuković M, Selthofer R, Vujić B, Ugljarević M. Successful of the B-Lynch compression suture in the management of massive postpartum hemorrhage: case reports and review. Arch Gynecol Obstet. 2006;273:307-9. <https://doi.org/10.1007/s00404-005-0059-1>

[21] Hossain N, Shansi T, Haider S, Soomro N, Khan NH, Memon GU, et al. Use of recombinant activated factor VII for massive postpartum hemorrhage. Acta Obstet Gynecol Scand. 2007;86:1200-6. <https://doi.org/10.1080/00016340701619324>

[22] Ilić-Mostić T, Sparić R, Argirović R, Ljubić A, Bozanović T, Arsenijević L, et al. [Our experince with the use of recombinant activated factor VII in postpartum haemorrhage]. Srp Arh Celok Lek. 2008;136 Suppl 3:204-9.

[23] James AH, Paglia MJ, Gernsheimer T, Grotegut C, Thames B. Blood component therapy in postpartum hemorrhage. Transfusion. 2009;49:2430-3. <https://doi.org/10.1111/j.1537-2995.2009.02318.x>

[24] Kalina M, Tinkoff G, Fulda G. Massive postpartum hemorrhage: recombinant factor VIIa use is safe but not effective. Del Med J. 2011;83:109-13.

[25] Kallianidis AF, Maraschini A, Danis J, Colmorn LB, Deneux-Tharaux C, Donati S, et al. Management of major obstetric hemorrhage prior to peripartum hysterectomy and outcomes across nine European countries. Acta Obstet Gynecol Scand. 2021;100:1345-54. <https://doi.org/10.1111/aogs.14113>

[26] Kamijo K, Nakajima M, Shigemi D, Kaszynski RH, Ohbe H, Goto T, et al. Resuscitative endovascular balloon occlusion of the aorta for life-threatening postpartum hemorrhage: A nationwide observational study in Japan. J Trauma Acute Care Surg. 2022;93:418-23. <https://doi.org/10.1097/ta.0000000000003650>

[27] Kavak SB, Kavak E, Demirel I, Ilhan R. Double-balloon tamponade in the management of postpartum hemorrhage: a case series. Ther Clin Risk Manag. 2014;10:615-20. <https://doi.org/10.2147/tcrm.S62574>

[28] Kayem G, Kurinczuk JJ, Alfirevic Z, Spark P, Brocklehurst P, Knight M. Specific second-line therapies for postpartum haemorrhage: a national cohort study. BJOG. 2011;118:856-64. <https://doi.org/10.1111/j.1471-0528.2011.02921.x>

[29] Kwong LT, So PL, Wong SF. Uterine compression sutures with additional hemostatic procedures for the management of postpartum hemorrhage. J Obstet Gynaecol Res. 2020;46:2332-9. <https://doi.org/10.1111/jog.14426>

[30] Lavigne-Lissalde G, Aya AG, Mercier FJ, Roger-Christoph S, Chauleur C, Morau E, et al. Recombinant human FVIIa for reducing the need for invasive second-line therapies in severe refractory postpartum hemorrhage: a multicenter, randomized, open controlled trial. J Thromb Haemost. 2015;13:520-9. <https://doi.org/10.1111/jth.12844>

[31] Lim G, Kotsis E, Zorn JM, Dalby PL, Ralph CJ, Waters JH. Cell salvage for postpartum haemorrhage during vaginal delivery: a case series. Blood Transfus. 2018;16:498-501. <https://doi.org/10.2450/2017.0155-17>

[32] Makino S, Takeda S, Kobayashi T, Murakami M, Kubo T, Hata T, et al. National survey of fibrinogen concentrate usage for post-partum hemorrhage in Japan: investigated by the Perinatology Committee, Japan Society of Obstetrics and Gynecology. J Obstet Gynaecol Res. 2015;41:1155-60. <https://doi.org/10.1111/jog.12708>

[33] Mouna K, Fehmi F, Kais OG, Amal N, Noura C, Amine BM, et al. Abstract P-12. Proceedings of Réanimation 2018, the French Intensive Care Society International Congress. Annals of Intensive Care. 2018;8:32. <https://doi.org/10.1186/s13613-017-0345-7>

[34] Mousa HA, Cording V, Alfirevic Z. Risk factors and interventions associated with major primary postpartum hemorrhage unresponsive to first-line conventional therapy. Acta Obstet Gynecol Scand. 2008;87:652-61. <https://doi.org/10.1080/00016340802087660>

[35] Murakami M, Kobayashi T, Kubo T, Hata T, Takeda S, Masuzaki H. Experience with recombinant activated factor VII for severe post-partum hemorrhage in Japan, investigated by Perinatology Committee, Japan Society of Obstetrics and Gynecology. J Obstet Gynaecol Res. 2015;41:1161-8. <https://doi.org/10.1111/jog.12712>

[36] Obata S, Kasai M, Kasai J, Seki K, Sekikawa Z, Torimoto I, et al. Emergent uterine arterial embolization using N-butyl cyanoacrylate in postpartum hemorrhage with disseminated intravascular coagulation. Biomed Res Int. 2017;2017:1562432. <https://doi.org/10.1155/2017/1562432>

[37] Palacios-Jaraquemada J, Fiorillo A. Conservative approach in heavy postpartum hemorrhage associated with coagulopathy. Acta Obstet Gynecol Scand. 2010;89:1222-5. <https://doi.org/10.3109/00016349.2010.491524>

[38] Park SC, Yeom SR, Han SK, Jo YM, Kim HB. Recombinant activated factor VII as a second line treatment for postpartum hemorrhage. Korean J Crit Care Med. 2017;32:333-9. <https://doi.org/10.4266/kjccm.2016.00787>

[39] Pasquier P, Gayat E, Rackelboom T, La Rosa J, Tashkandi A, Tesniere A, et al. An observational study of the fresh frozen plasma: red blood cell ratio in postpartum hemorrhage. Anesth Analg. 2013;116:155-61. <https://doi.org/10.1213/ANE.0b013e31826f084d>

[40] Pellerin O, Bats AS, Di Primio M, Palomera-Ricco A, Pinot de Villechenon G, Fournier L, et al. Postpartum hemorrhage treated with gelfoam slurry embolization using the superselective technique: immediate results and 1-month MRI follow-up. Cardiovasc Intervent Radiol. 2013;36:98-104. <https://doi.org/10.1007/s00270-012-0355-7>

[41] Phillips LE, McLintock C, Pollock W, Gatt S, Popham P, Jankelowitz G, et al. Recombinant activated factor VII in obstetric hemorrhage: experiences from the Australian and New Zealand Haemostasis Registry. Anesth Analg. 2009;109:1908-15. <https://doi.org/10.1213/ANE.0b013e3181c039e6>

[42] Phillips J, Tamura T, Sakamoto S, Waters J. Autotransfusion during postpartum hemorrhage following vaginal delivery. Anesth Analg. 2020;131:10. <https://doi.org/http://dx.doi.org/10.1213/01.ane.0000696764.47378.76>

[43] Ramler PI, van den Akker T, Henriquez D, Zwart JJ, van Roosmalen J. Incidence, management and outcome of women requiring massive transfusion after childbirth in the Netherlands: secondary analysis of a nationwide cohort study between 2004 and 2006. BMC Pregnancy Childbirth. 2017;17:197. <https://doi.org/10.1186/s12884-017-1384-7>

[44] Ramler PI, Henriquez D, van den Akker T, Caram-Deelder C, Groenwold RHH, Bloemenkamp KWM, et al. Comparison of outcome between intrauterine balloon tamponade and uterine artery embolization in the management of persistent postpartum hemorrhage: A propensity score-matched cohort study. Acta Obstet Gynecol Scand. 2019;98:1473-82. <https://doi.org/10.1111/aogs.13679>

[45] Sentilhes L, Gromez A, Razzouk K, Resch B, Verspyck E, Marpeau L. B-Lynch suture for massive persistent postpartum hemorrhage following stepwise uterine devascularization. Acta Obstet Gynecol Scand. 2008;87:1020-6. <https://doi.org/10.1080/00016340802380750>

[46] Sharma R, Sirsam S, Koranne P, Wahane A. Balloon tamponade—a novel innovation in the management of refractory postpartum hemorrhage at tertiary care center: a study from central India. JSAFOG. 2021;13:221-5. <https://doi.org/10.5005/jp-journals-10006-1930>

[47] Sobieszczyk S, Breborowicz GH, Platicanov V, Tanchev S, Kessler CM. Recombinant factor VIIa in the management of postpartum bleeds: an audit of clinical use. Acta Obstet Gynecol Scand. 2006;85:1239-47. <https://doi.org/10.1080/00016340600855839>

[48] Stein W, Spätling L. Effect of early "quilting" sutures on morbidity in postpartum hemorrhage. Int J Gynaecol Obstet. 2019;144:62-6. <https://doi.org/10.1002/ijgo.12700>

[49] Stensaeth KH, Sovik E, Haig IN, Skomedal E, Jorgensen A. Fluoroscopy-free resuscitative endovascular balloon occlusion of the aorta (REBOA) for controlling life threatening postpartum hemorrhage. PLoS One. 2017;12:e0174520. <https://doi.org/10.1371/journal.pone.0174520>

[50] Thurn L, Wikman A, Lindqvist PG. Postpartum blood transfusion and hemorrhage as independent risk factors for venous thromboembolism. Thromb Res. 2018;165:54-60. <https://doi.org/10.1016/j.thromres.2018.03.002>

[51] Touhami O, Marzouk SB, Kehila M, Bennasr L, Fezai A, Channoufi MB, et al. Efficacy and safety of pelvic packing after emergency peripartum hysterectomy (EPH) in postpartum hemorrhage (PPH) setting. Eur J Obstet Gynecol Reprod Biol. 2016;202:32-5. <https://doi.org/10.1016/j.ejogrb.2016.04.013>

[52] Wassim F, Bilel BJ, Fedi S, Hayfa H, Ines A, Hayfa R, et al. Abstract P-31. Proceedings of Réanimation 2018, the French Intensive Care Society International Congress. Annals of Intensive Care. 2018;8:32. <https://doi.org/10.1186/s13613-017-0345-7>

[53] Wikkelsø AJ, Edwards HM, Afshari A, Stensballe J, Langhoff-Roos J, Albrechtsen C, et al. Pre-emptive treatment with fibrinogen concentrate for postpartum haemorrhage: randomized controlled trial. Br J Anaesth. 2015;114:623-33. <https://doi.org/10.1093/bja/aeu444>

[54] WOMAN trial collaborators. Effect of early tranexamic acid administration on mortality, hysterectomy, and other morbidities in women with post-partum haemorrhage (WOMAN): an international, randomised, double-blind, placebo-controlled trial. Lancet. 2017;389:2105-16. <https://doi.org/10.1016/s0140-6736(17)30638-4>

[55] Zainab F, Ali S, Khakwani M. Audit of patients undergoing obstetrical hysterectomy for postpartum hemorrhage. PJMHS. 2015;9:3.

[56] Downs SH, Black N. The feasibility of creating a checklist for the assessment of the methodological quality both of randomised and non-randomised studies of health care interventions. J Epidemiol Community Health. 1998;52:377-84. <https://doi.org/10.1136/jech.52.6.377>
